# Learning in the workplace: Use of informal feedback cues in doctor‐patient communication

**DOI:** 10.1111/medu.14148

**Published:** 2020-04-20

**Authors:** Carolin Sehlbach, Pim W. Teunissen, Erik W. Driessen, Sharon Mitchell, Gernot G. U. Rohde, Frank W. J. M. Smeenk, Marjan J. B. Govaerts

**Affiliations:** ^1^ Department of Educational Development and Research School of Health Professions Education Faculty of Health, Medicine and Life Sciences Maastricht University Maastricht the Netherlands; ^2^ Department of Obstetrics and Gynaecology Amsterdam University Medical Centre Amsterdam the Netherlands; ^3^ World Heart Federation Geneva Switzerland; ^4^ Department of Respiratory Medicine University Hospital Goethe University Frankfurt am Main Germany; ^5^ Department of Respiratory Medicine Catharina Hospital Eindhoven the Netherlands

## Abstract

**Objectives:**

We expect physicians to be lifelong learners. Participation in clinical practice is an important potential source of that learning. To support physicians in this process, a better understanding of how they learn in clinical practice is necessary. This study investigates how physicians recognise and use informal feedback from interactions with patients in outpatient settings as learning cues to adjust their communication behaviours in daily practice.

**Methods:**

To understand physicians’ use of informal feedback, we combined non‐participant observations with semi‐structured interviews. We enrolled 10 respiratory physicians and observed 100 physician‐patient interactions at two teaching hospitals in the Netherlands. Data collection and analysis were performed iteratively according to the principles of constructivist grounded theory.

**Results:**

Following stages of open, axial and selective coding, we were able to conceptualise how physicians use cues to reflect on and adjust their communication. In addition to vast variations within and across patient encounters, we observed recurring adjustments in physicians’ communication behaviours in response to specific informal feedback cues. Physicians recognised and used these cues to self‐monitor communication performance. They had established ‘communication repertoires’ based on multiple patient interactions, which many saw as learning opportunities contributing to the development of expertise. Our findings, however, show differences in physicians’ individual levels of sensitivity in recognising and using learning opportunities in daily practice, which were further influenced by contextual, personal and interpersonal factors. Whereas some described themselves as having little inclination to change, others used critical incidents to fine‐tune their communication repertoires, and yet others constantly reshaped them, seeking learning opportunities in their daily work.

**Conclusions:**

There is large variation in how physicians use learning cues from daily practice. To enhance learning in and from daily practice, we propose turning workplace learning into a collaborative effort with the aim of increasing awareness and the use of informal performance‐relevant feedback.

## Abstract 2

Sehlbach et al. use observations and interviews to examine how physicians use informal feedback for professional development, highlighting how workplace learning could be improved through collaboration.

**Key message**

There is large variation in how physicians use cues from daily patient interactions as informal feedback, to adjust their communication and learn from daily practice.

## 1 INTRODUCTION

Certified physicians are expected to engage in lifelong learning. As such, they are often required to prove their participation in formal learning activities, such as courses or congresses, which too often are didactic and primarily knowledge‐based.^1^, ^2^, ^3^ In fact, research findings suggest that most physician learning occurs informally through work.^4^, ^5^ Learning can then be conceptualised as the individual, situated and social process of giving meaning to (various) personal experiences that contribute to ongoing personal and professional development. This process often leads to ‘reinforcing or only slightly adjusting knowledge or behaviour’ and sometimes to an ‘observable (and potentially significant) change in future performance.’^6^ Not all learning, however, leads to an observable change. Compared with formal learning activities, informal learning is mostly unplanned, unconscious or tacit, and involves others through meaningful experiences in an authentic setting.^7^, ^8^ Informal learning can also be deliberate when physicians consciously aim to improve performance by monitoring their own performance, reflecting on their own behaviour and its effect, and seeking feedback for learning.^9^ The process of self‐monitoring and reflection on one's own behaviour and experiences in clinical practice is thus important to optimise (future) performance.^4^, ^9^, ^10^, ^11^, ^12^ Making informal learning more explicit and deliberate may, therefore, enhance physicians’ lifelong learning.^4^, ^13^ This specifically applies to the further development and refinement of physicians’ communication skills. Good communication is the keystone of a fruitful doctor‐patient relationship and high‐quality care. Communication is a complex yet crucial skill^14^ that is predominantly developed through practice and informal learning.

Although feedback and reflection on interactions with patients or peers in the workplace may stimulate physicians’ learning, it is well noted that certified physicians receive little formal feedback. Yet, informal feedback through other ‘learning cues’ including, for example, patient responses, clinical outcomes and conversations with colleagues, specifically reported by Watling and colleagues,^15^ may ‘facilitate the interpretation of the experience and the construction of knowledge from it.’^15^, ^16^, ^17^ However, it may be challenging for physicians to recognise those cues as cues for learning because they can be ambiguous and are strongly embedded in daily routine.^4^ In day‐to‐day practice, physicians predominantly engage with patients using their routine expertise, and are less involved with their workplace learning.^12^ They may therefore benefit from support in recognising meaningful learning cues, as well as in knowing how to use and learn from them, so that they might consider adapting and improving their practice.^2^, ^13^, ^15^, ^16^, ^17^

A better understanding of how physicians interpret meaningful experiences and use cues from daily practice as informal feedback may support strategies for physicians to develop their expertise, essential to ensuring the provision of high‐quality care.^18^, ^19^ We, therefore, aim to further refine theory on how physicians learn in the workplace setting from day‐to‐day experiences in patient care.^18^ More specifically, we investigate how physicians recognise and reflect on learning cues related to their communication with patients and, consequently, learn and adjust their practice through patient interactions.

## 2 METHODS

We used non‐participant observations and semi‐structured interviews to explore physicians’ use of informal feedback cues in doctor‐patient communication, with the overarching aim of refining our conceptual understanding of workplace learning in patient care. Data collection and data analyses were informed by principles of constructivist grounded theory (CGT).^20^, ^21^ Throughout the collection and interpretation of our data, we specifically acknowledged team members’ theoretical and practical knowledge of doctor‐patient communication, (workplace‐based) learning and feedback.

### 2.1 Setting and participants

We approached physicians in two teaching hospitals in the Netherlands, combining purposeful and theoretical sampling. To ensure that we sampled a range of work settings with different levels of care, we approached one hospital that primarily focuses on secondary care and one that also delivers tertiary care. To ensure homogeneity with respect to the area of patient care, we selected respiratory medicine specialists working with outpatients in respiratory disease clinics.^22^ Respiratory medicine covers a varied patient population including acute, chronic and terminally ill patients, which often results in the development of long‐standing and intensive physician‐patient relationships that offer unique opportunities to explore our research question. We purposefully sampled respiratory medicine specialists with variation in age, gender, subspecialisation and experience. After the first round of data analysis, the research team decided to include additional physicians who were at the beginning or in the middle of their careers because the first interviewees were more senior.^22^ All physicians consented to participate.

Upon registration for outpatient appointments, patients were sent a short letter, which informed them about the research and the presence of a researcher as an observer (CS), and were asked for consent. The participating physicians verbally briefed each individual patient, emphasising that the research focused on the physician, that no patient data would be collected, and that CS did not have a medical background. If, after the consultation had started, either the physician or patient preferred the researcher not to be present, CS left the room.

We obtained ethical approval from the Netherlands Association for Medical Education (NVMO: file no. 2018.7.9), and from the ethical committees of both participating hospitals (file nos 2018‐0864 and nWMO‐2018.118).

### 2.2 Data collection

We combined data from non‐participatory observations with data from informal and semi‐structured interviews in an iterative design. CS shadowed 100 appointments of 10 physicians in outpatient clinics. CS observed physician‐patient encounters and what cues physicians seemed to react to. CS particularly focused on variations in communication style during and across consultations and on if, how and when physicians changed their communication with patients. Observed adaptations in behaviour were used during the subsequent interview to probe which feedback cues physicians responded to and whether observations reflected conscious adaptations in behaviour. During the observations, CS took field notes, which she worked out within the 48 hours after the observation to prevent loss of information. Observations lasted 1.5‐3.5 hours, during which CS usually observed eight to 12 outpatient appointments (Table 1). CS sat at some distance from the physician and patient, and asked participating physicians to conduct the clinic following a typical daily routine.

**Table 1:** Overview of physician‐patient interactions observed

Interviewee	Patient encounters, n	Hours observed, n
A1	12	3
A2	8	2.5
A3	11	3.5
A4	11	2.5
A5	12	3
A6	11	3
A7	11	3
A8	10	3
A9	10	3
A10	4^a^	1.5
Total	100	28

^a^ One observation included four patient encounters in a highly specialised outpatient clinic for patients with a certain rare disease.

After the observations, CS used her notes to prepare interview questions on the situations observed. Following this preparation (0.5‐1.5 hours), CS conducted semi‐structured interviews (Appendix S1). During the interviews, physicians considered and reflected on the cues they used to adjust their communication. First, CS inquired about changes she had observed in physicians’ non‐verbal or verbal communication during specific patient encounters in order to understand whether that change was a deliberate process of sense making and reflection. Second, CS posed questions that addressed differences in how physicians approached different patients. Drawing on these observations, CS probed the physicians interviewed on their awareness of the learning cues that had led them to alter communication during interactions with patients and the process of giving meaning to those cues. The semi‐structured interviews lasted 30‐55 minutes, and were recorded and transcribed verbatim. With the help of the transcriptions, notes were transformed into concrete reconstructions with analytic memos and commentaries.^21^ The data were subsequently analysed to obtain an in‐depth understanding of the cues physicians considered relevant to their communication, and how they used these cues for learning.

### 2.3 Data analysis

Data collection and data analysis were performed in an iterative manner so that early analysis influenced the focus of subsequent observations and interviews. CS started by open, line‐by‐line coding of three interviews and the corresponding observational field notes. The authors FWJMS, EWD, PWT, GGUR and MJBG each read and coded one interview transcript and related field notes and discussed their respective codes with CS independently. After refining the coding framework, the research team discussed and collated the codes into preliminary categories. PWT and CS jointly discussed these, which resulted in the following preliminary categories: context; physician‐patient relationship; patient characteristics; anticipatory or reactive change in communication, and routine. CS used the preliminary categories in the axial coding of two more interview transcripts before discussing categories with SM and PWT separately to identify relationships between categories. This led to the construction of the following additional categories: external factors; examples of learning cues (patient reaction, physician reaction), reflection, and communication repertoire. CS and SM conceptualised the categories into a conceptual model, based on which CS coded three more interviews. CS, SM, FWJMS, EWD and MJBG discussed and agreed on the conceptual model, and further deliberated how contextual factors affected physicians’ decisions to react to and learn from cues or not. After CS had coded the two remaining transcripts, the research team re‐examined the conceptual model and adjusted it into a final conceptual model of how physicians recognised and used informal feedback cues for their learning and how the use of cues resulted in a change in communicative behaviour (selective coding).

We collected and analysed data until we felt that no new concepts came up and that we had reached theoretical sufficiency to answer the research question and to build theory.^23^ Data were managed with atlas.ti (Scientific Software Development GmbH, Berlin, Germany) and the analysis was reported with the COREQ (consolidated criteria for reporting qualitative research) checklist (Appendix S2).

### 2.4 Reflexivity

The research team maintained reflexivity throughout data collection, data analysis and the process of writing up the results by discussing underlying assumptions about physician‐patient communication and learning cues. CS used reflective memos throughout data collection to reflect on how her own experiences and pre‐constructed knowledge influenced the interpretation of findings. These helped her to reflect on how her interactions with participants, her presence during the consultations and her beliefs about the construction of knowledge through interactions affected the way she looked at the data. CS has a background in health sciences and is a PhD student in medical education, focusing on physicians’ lifelong learning.

The research team also consisted of medical educators (MJBG and EWD) and medical specialists (FWJMS, GGUR and PWT). FWJMS and GGUR are experienced respiratory medicine specialists and each has their own experiences and set of values regarding good communication with respiratory disease patients. PWT is specialised in gynaecology and obstetrics and has expertise in workplace‐based learning. PWT’s beliefs on learning through experiences and ideas on informal feedback most certainly affected the way he interpreted the data, as did his experiences in communication with patients. SM is a medical education manager with her own beliefs about learning. All members of the research team (CS, PWT, EWD, SM, GGUR, FWJMS and MJBG) are involved in medical education and have a particular focus on workplace learning, continuing education or performance assessment. Their respective backgrounds may have influenced the research in presuming that physicians learn from daily patient encounters. The combination of medical and non‐medical backgrounds and various interests resulted in repeated discussion on how team members analysed and interpreted the data based on their own beliefs. These deliberations enriched our process of interpreting the data and constructing themes.

## 3 RESULTS

Our analysis revealed differences in how physicians reacted to, reflected on and learned from various cues derived from outpatient communication. Table 2 shows potential cues identified by participants and observed by the researcher.

**Table 2:** Examples of different cues in patient interactions

Cues physicians may react to:	Examples
Circumstantial cues	Time constraints Goal of the outpatient appointment
Environmental cues	Physical set‐up of the room
Patient characteristics	Diagnosis, disease status, physical condition, age, gender, education, mood, values and beliefs
Personal cues	Physician's mood, values and beliefs
Interpersonal cues	Previously established physician‐patient relationship

**Figure 1:**
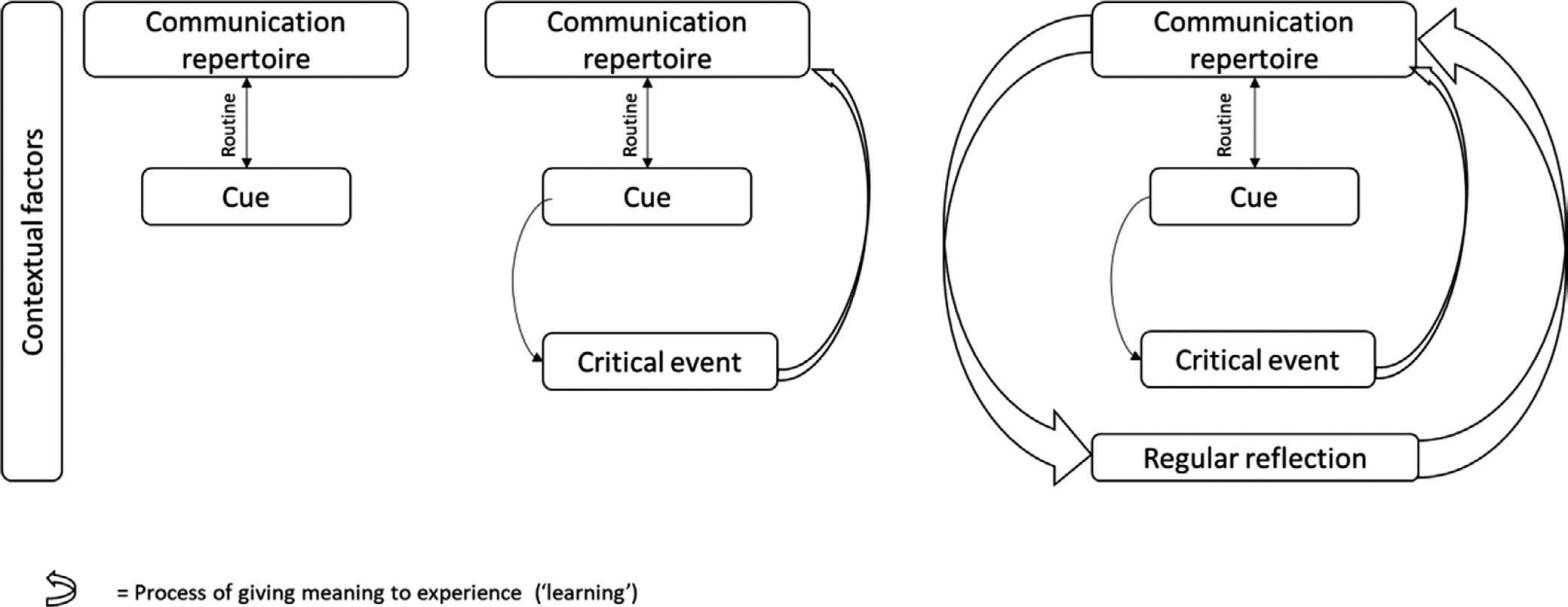
Conceptual model

Based on their extensive experiences of communicating with patients, physicians had built their own communication repertoire. When self‐monitoring task performance (ie, communication with patients), physicians recognised and used informal cues to frequently adjust their communication behaviours, either consciously or automatically. Performance feedback that was interpreted as ‘critical’ was likely to result in significant changes in the communication repertoire, or, in case of a positive experience, in its reinforcement for future use. It is these processes of giving meaning to and reflecting on experiences during and after task performance that reflect how physicians learn through and from their work. Figure 1 visually depicts these processes. Participants, however, differed in their sensitivity to informal cues, influenced by contextual factors. They also differed in their recognition of the learning that resulted from their practice.

We first describe how physicians adjusted their communicative behaviours within and across outpatient appointments in reaction to cues, as part of their routine. Subsequently, we report how physicians felt they used informal feedback as learning opportunities in their current practice.

### 3.1 Variation in communication within and between patient encounters

Physicians faced a large variety of interactions in the outpatient setting. We noticed recurring behaviours in physicians’ communication, which they indicated to have acquired from previous interactions.

Physicians’ communication behaviours, however, changed within and between patient encounters. This often happened unconsciously and automatically. They continuously adjusted to patients’ reactions and circumstantial cues, smoothly manoeuvring through different patient encounters. One physician portrayed this as ‘the game in the consulting room’ (Observational field notes, A7). They had a ‘communication repertoire’ at their disposal, which guided them through their daily routine as they either deliberately or unconsciously reacted to context‐specific cues:

It is actually just one strategy … that is, responding to what the patient gives you back. That can be really easy sometimes and passes very quickly, but other times you really need to manoeuvre carefully. … I now have a huge repertoire of standard reactions that I can draw on … (Interview, A4)

Our interviews forced physicians to deliberate on the preceding patient encounters and potential learning opportunities. They were often unconscious of their communication repertoires and how they used similar strategies across patients:

How funny. … It could be. … I didn't know that I pushed my chair back, but now that you mention it, I think, oh yeah, that's right, I do that. (Interview, A8)

Interviewees repeatedly mentioned that an absence of patient complaints implied that their communication repertoire was sufficient and did not require further development. They heavily relied on the repertoire they had built and believed they could ‘no longer be corrected’ (Interview, A5). Despite comprehending its importance, physicians hardly dwelt on their communication during or after patient encounters. Some struggled to pinpoint how they adapted their communication throughout patient encounters, which reflects an automatic behavioural adjustment. Others knew they used humour in their consultation but often mentioned it as an unintentional and automated strategy:

That way you try to keep it a bit personal, to put people at ease perhaps. … But that is something that you sense unconsciously. It’s not as if I think like ‘Oh let’s make a joke now,’ it’s something that happens unknowingly, actually. (Interview, A10)

Physicians’ communication repertoires included, but were not limited to, different strategies of taking the lead in the conversation, drawing to illustrate or explain a diagnosis, prognosis or treatment, and making changes in body language and non‐verbal communication. Some observations showed, for instance, that physicians leaned forward to show interest in and empathy for the patient or to emphasise the severity of a patient's condition. Some used physical contact to comfort or console patients by briefly touching an emotional patient's hand or patting the patient on the back when saying goodbye. Similarly, physicians signalled the end of a consultation by leaning backward, pushing back their chair or standing up.

Our participants realised that different factors, such as the atmosphere or goal of the consultation, the physical space of the clinic and time pressure, added complexity to outpatient appointments. Recognising these circumstantial cues, they understood that patients might be agitated when coming for a test result or check‐up after a treatment and had learned to adjust their communication accordingly. One interviewee, for example, gauged the atmosphere during a patient encounter in order to decide, deliberately, whether to open or wrap up a consultation or to reassure the patient with a joke:

[The physician jokes:] With this, you should be able to live to be 100. But I cannot give you any guarantee, of course. (Observation field notes, A4)

The same physician had made identical, age‐related jokes in previous consultations. In another consultation, however, he remained distant and refrained from making jokes because, as he explained afterwards, ‘This was not the most cheerful patient.’ These observations underline the finding that, depending on the circumstances, physicians also adapted their communication deliberately. Along with circumstantial cues such as atmosphere, workload and time constraints, interpersonal and personal cues also affected physician‐patient communication, including the relationship previously established, and the physician’s and patient’s mood, as well as their norms, values and attitudes:

You only really get to know people if you see them often. … At a regular check‐up … I do think that when you see familiar people … that I pay more attention to ‘What kind of person was that again?’ … That is, I think, your mental preparation, and I think at that point you are already largely determining your communication strategy. (Interview, A2)

We observed how physicians adjusted their communication when anticipating their patient’s reaction by slowing down, leaving a silence, using an increasing amount of gestures or giving more detailed information. We were able to distinguish between ad hoc adjustments in reaction to an unexpected turn of events, such as a patient bursting into tears or a phone ringing, and planned behaviour in anticipation of the consultation flow (ie, breaking bad news), as in the observed consultation described below:

A cancer patient and his partner return to discuss treatment options because their son had expressed his dissatisfaction about the diagnosis and treatment option the trainee had given his father. The trainee asks the attending physician to lead the consultation and to discuss the diagnosis and treatment plan with the patient and his partner.

The physician explains the diagnosis. He speaks more calmly than before when listing treatment options, pauses and gesticulates with his hands. ‘Let me draw it. You will receive more information about this later, but schematically for now …’

The phone rings twice during the consultation. The physician silences it both times, the second time even without looking at the phone or pausing his explanations. When there is a third call, the trainee takes it.

The phone also rang during other consultations that day, but this time the physician did interrupt the ongoing conversation to answer it. (Observation field notes, A7)

### 3.2 Learning from daily practice

Reflecting on their communication helped physicians realise how they had acquired their communication repertoire and developed their expertise in this domain. Our analysis of the observation and interview data identified various ways in which physicians recognised and used informal cues from past or present patient encounters as learning opportunities to change or optimise their communication repertoire in daily practice. Physicians differed in how they recognised and used informal feedback cues. Although all physicians had acquired a daily routine, some were able to uncover learning opportunities within this routine and engaged in ongoing expertise development. However, other physicians, mostly more senior physicians, felt that they were no longer able or willing to learn or adjust. They felt that time constraints and daily routine took priority over using patient encounters as learning opportunities to adapt their communication. They described themselves as ‘stuck in this pattern’ (Interview, A5), which prevented them from learning communicative subtleties, and commented that the ever‐repeating routine had decreased their sensitivity and willingness to learn from their interactions with patients.

Even stronger, many considered their communication repertoire as part of their personality:

That's how I am. Some [physicians] are more cheerful or more business‐like. It's not something you can switch on or off. (Interview, A1)

One of our respondents explained:

If this is the right way or [if] it could be better, yes, that may be so, but this is *my* way. And I don't think that I’m doing it a lot differently now from the way I did it 10, 20 or 30 years ago. (Interview, A5)

For this experienced physician and most of his colleagues, ongoing learning did not represent a smooth learning curve, but occurred intermittently and was induced by emotion or critical events:

You know, frankly, that sounds very arrogant, but I don't think I'm doing a very bad job. But I know for sure that sometimes I can be entirely wrong. And I do try to learn from that. It touches me. It certainly touches me. (Interview, A5)

Many physicians indicated that they learned from difficult or peculiar patient encounters, which were beyond the scope of our observations. They reported that those learning events often stood out from daily routine and that they:

[ranged] from someone who [is holding] a chair above his head and threatening you, so to speak, to people who … well, all sorts of variations. (Interview, A7)

Critical incidents resulted in increased reflection, which made physicians question their own behaviour and re‐evaluate their communication repertoire. One of our physicians painfully described his communication as a shortcoming because his patient continued smoking after repeated warnings. He believed himself to ‘be failing as a doctor’ (Interview and Observational field notes, A6) and recognised that he needed to learn how to deal with patients who trivialise their conditions. This shows that despite having developed reliable repertoires, physicians’ learning behaviours may change when their experiences elicit emotions, especially when they feel they have failed.

Other participants, however, internalised regular reflection as part of practising, continuing improvement and learning from their practice. These physicians readily acknowledged that their communication repertoire was ‘based on previous experiences’ (Interview, A4). They were aware of the constant yet subtle adaptations in their behaviour within and between patient encounters. Physicians used these to play ‘the game in the consulting room’ of deliberately fine‐tuning and mastering their communication repertoires:

You try to act on the patient's level. This morning, we had that farmer, right, that man who reacts primarily driven by his emotions with a certain rigidity and then you try to tune into that level. (Interview, A7)

Some indicated that reflection on and learning from patient encounters had become fully ingrained in their daily practice:

I’m always, I think, learning a bit anyway. (Interview, A8)

As someone explained in reaction to our observations:

Yes, I do think that I actually always take note, when the patient [is] in the consulting room or [when I] start preparing for the next patient. Like, … for this patient, but also for similar cases, … how do I say that in plain and clear language? … I did learn from this and reflected [on it] again and that’s actually how I do it every time. (Interview, A2)

## 4 DISCUSSION

We aimed to explore how physicians use cues from their clinical practice as informal feedback to adjust their communication and learn from daily patient interactions. Our data showed variation in physicians’ communication within and across outpatient encounters influenced by personal, interpersonal and contextual factors. Physicians reported that they had learned from previous interactions, which established communication repertoires that guided them through their practice. Our findings show different degrees in the extent to which physicians monitored their behaviour, and recognised and used informal feedback from daily practice for their learning.

Some physicians were less likely to engage in reflection and deliberate learning from day‐to‐day patient care, whereas others appeared more sensitive to learning cues and engaged in ongoing reflection and improvement.

According to Ericsson's model of deliberate practice and expert performance,^10^ physicians who indicated that they felt themselves to be caught up in the demands of daily practice and did not have the time or willingness to reflect on their communication repertoire could be considered to be in a phase of arrested development. Yet, these physicians felt competent in their performance and would potentially reinitiate a learning process in response to critical events. Others continuously challenged their communication repertoire to ultimately increase their control over unfamiliar and challenging situations.^24^ They reflected on whether they had used their repertoires effectively and efficiently, which allowed them to navigate smoothly through practice and indicated their mastery and expert performance.^9^ These findings are in line with how Archer describes the influence of ‘the self’ in relation to the self‐monitoring of performance and use of (external) feedback. He argues that together with its format, self‐awareness, acceptability and the recipient's goal setting affect the use of feedback.^25^

The ways in which participants in our study described and reflected on the development and use of their communication repertoires reflect findings from a recent study on how trainees develop expertise in communication.^26^ Kawamura et al^26^ distinguish between procedural fluency and conceptual understanding in expertise development in communication. The use of humour or physical contact to comfort a patient can be considered procedural fluency, whereas ‘shifting’ between patients and moving towards the patient’s ‘level’ reflect a physician's conceptual understanding of distinct patient needs.^26^ Adaptive experts thus command a conceptual understanding of when they should use their established communication repertoire and when they should adapt the way they communicate their clinical knowledge to different patients or innovate strategies for adapting to non‐routine situations. Findings from our study seem to confirm Kawamura and colleagues’ conclusion that learning how to ‘shift’ between patients is key to the development of expertise in communication.^26^ Our findings also raise questions about, which factors influence physicians’ positions on the learning curve, and how we can create a work environment that stimulates all physicians to become lifelong learners.

Contextual factors within daily practice influenced physicians’ willingness and sensitivity to recognise learning opportunities. Factors such as workload and time pressure in the clinical workplace may impede physicians’ reflection on learning cues and their learning.^17^, ^27^, ^28^, ^29^ Our work may aid in identifying ‘contextual factors that influence self‐monitoring behaviours in the moment of action.’^11^ Some of our participants were more sensitive to time pressure and more preoccupied with daily routine than others, which may explain the individual differences in how deliberately our participants recognised learning opportunities. These findings align with the findings of Kyndt and colleagues,^28^ who suggest that learner characteristics, as well as organisation type and size, determine the acknowledgement of learning opportunities at work. They also resonate with what Billet describes as the setting's readiness to afford learning opportunities.^7^ This hints at possibilities for improving the overall learning climate in hospital departments.^30^, ^31^ Engaging physicians in lifelong learning requires a culture, which proactively stimulates reflection and which recognises the tension between performance and continuous learning. When physicians are given opportunities to reflect more on their communicative behaviour during patient care, through formal and informal feedback, learning may be supported and stimulated. Engaging in these types of learning activity in workplace settings is likely to outperform formal educational activities in terms of effectiveness.^11^, ^24^

### 4.1 Implications in relation to lifelong learning

Our results indicate that we may need to support physicians in engaging in reflection in and on action, as well as being cautious of potentially negative effects. We need to balance routine practice with reflective practice (to support the development and maintenance of expert performance) as continuous reflection may hinder physicians in delivering care.^9^ That is, physicians need to have a certain level of automaticity in order to practise in the context of increasing practice demands and contextual factors such as workload and time pressure.

Nonetheless, if feedback and reflection are truly valued as essential attributes for lifelong learning, more importance must be placed upon physicians’ awareness of their own behaviour during practice, combined with a willingness to learn in order to improve. The UK revalidation system, as one of few national recertification systems, already requires physicians to reflect on critical incidents, feedback received, and compliments or complaints during an annual appraisal. Although this creates workplace‐based opportunities for physician learning, physicians are likely to reflect on aggregated data only, de‐emphasising learning in context. Rather, if we want to stimulate more reflective behaviour in practice, we may consider making a collective effort to create an environment that stimulates learning. Physicians could, for instance, occasionally observe peers in order to experience diverse communication approaches and thus to learn for their own practice, similarly to how trainees learn from observing faculty staff.^27^ To initiate collaborative learning, physicians could also engage in peer consultation and share experiences and recent learning as described by Mylopoulos et al as ‘discover then tell.’^32^ Those who are capable of discovering learning opportunities amongst patient interactions could guide colleagues who are less sensitive to cues in a collective effort to engage in deliberate practice.^9^ Perhaps some of these collective learning activities could then serve as evidence that a physician contributes to a learning environment and hence as a marker for recertification.

Although our suggestions for practical implications may sound idealistic, we do consider them as representing an investment in physicians’ performance improvement. Given the high numbers of patient complaints regarding physicians’ communication, such initiatives may well be worth the time and monetary investment.^33^

### 4.2 Strengths and limitations

A first limitation of the current study is that we based our data on single‐day observations and self‐perceived learning strategies. We did not observe the same physician for longer periods, which might have resulted in multiple observations of physicians’ learning behaviours, or outside the outpatient setting. Therefore, we were not able to actually investigate ‘learning’ as represented by persistent changes in performance. Based on our conceptualisation of learning, however, our findings reflect the ways in which physicians use informal feedback cues to give meaning to day‐to‐day experiences as a starting point for the reinforcement or adjustment of their communication repertoires.

Second, we collected data in outpatient clinics for respiratory disease, observing and interviewing respiratory medicine specialists. Both hospitals were teaching hospitals, although they provided different levels of care (mainly secondary or mainly tertiary care). This may present another limitation as physicians working in these clinics may have been more aware of and involved in others’ or their own learning as a result of their teaching roles. These physicians may have (developed) a more learning‐focused mindset than physicians without education duties. It may be worthwhile to explore how physicians learn informally in non‐teaching contexts.

Third, the focus of our study was communication. Hence, our findings on how physicians use learning cues may be specific to communication and whether the current findings are transferrable to learning in other competency domains, such as medical knowledge or technical skills, may be questionable. Additional research into the nature of informal feedback cues and physicians’ use of informal feedback in other competency domains might further our understanding of physicians’ workplace learning from daily working routine.

In the Netherlands, recertification procedures and requirements to guide physicians’ lifelong learning are similar across medical specialties. This makes our results transferable to other specialties, particularly considering that variations between and within individuals are presumably present across all specialties. Research in other specialties or clinical contexts and settings, however, may lead to different findings.

A strength of our study was its combination of non‐participant observations and interviews. Many others have previously recommended the use of this combination of methods to present data on what physicians report themselves, as well as on what can be observed.^11^, ^17^, ^18^, ^34^ The observation of physicians in the outpatient setting was instrumental in helping us to explore learned behaviour and learning opportunities in practice.

## 5 CONCLUSIONS

There is large variation in how physicians use learning opportunities in outpatient settings. Their informal learning is influenced by contextual, personal and interpersonal factors, which may either promote or inhibit physicians’ reflection and learning. Our findings suggest opportunities to increase physicians’ awareness of when and how they can learn from daily practice. To address the lack of awareness of learning in and from daily practice, we propose to turn learning into a collaborative effort. This requires a learning culture in the workplace, in which physicians can use existing differences between their own and their peers’ performance to learn from one another.

## AUTHOR CONTRIBUTIONS

CS collected and analysed the data, and drafted and revised the paper. PWT, EWD, FWJMS and MJBG contributed to the analysis of the data, and the drafting and revision of the paper. SM contributed to the interpretation of the data, and the drafting and revision of the paper. GGUR contributed to the drafting and revision of the paper. All authors (CS, PWT, EWD, SM, GGUR, FWJMS and MJBG) designed and conceived the study, jointly wrote the research plan, had full access to the data, take responsibility for the integrity and accuracy of the data analysis, and approved the final manuscript for submission.

## FUNDING INFORMATION

The European Respiratory Society funded this study. However, CS, who was appointed to the research project as a PhD student at Maastricht University, conducted the research independently of this funding. The European Respiratory Society had no role in the design or conduct of the study, the collection, analysis and interpretation of data, or the preparation or approval of the manuscript or the decision to submit for publication.

## CONFLICTS OF INTEREST

None.

## ETHICAL APPROVAL

This study was approved by the Netherlands Association for Medical Education (NVMO: file no. 2018.7.9), and the ethical committees of both participating hospitals (file nos 2018‐0864 and nWMO‐2018.118).

## Supporting information

Appendix S1

Appendix S2
