# Supplementary material for: Learning in the workplace: Use of informal feedback cues in doctor‐patient communication
Source: Med Educ. 2020 Apr 20;54(9):811–20. doi: 10.1111/medu.14148 (PMC7496915; doi:10.1111/medu.14148)
Supplement: Supplementary file 1 — Appendix S1 [file MEDU-54-811-s001.docx]

Appendix II

Interview protocol

Opening:

- For this research we want to investigate which information sources doctors use to gain insight into their functioning, in particular their communication with patients.
- How did you experience the today? What were your experiences with regard to communication and interaction with patients?
- What influences your communication in daily practice?
- Do you ever reflect on your daily functioning? When? How? Induced by what?

Key questions:

- Were there interactions / situations today that made you to aware of the way your communication? Or when did you alter the way you communicated?
  - Did you feel uncomfortable at any when communication with your patients? Or were there actually pleasant experiences?
    - Elaboration (what, how, why, etc)
- What did these experiences / emotions / reflection mean for you?
  - - Are you going to do something with this? If so what (Further training? Intervision?)

• What struck me today during my observation was [refer to a particular encounter/specific situation] what was relevant to you in this situation / interaction?

- - What were your assumptions when patient XY entered?

• What made you change the way you interact between patient A and patient B?

- - - When did you adjust the way you communicated? Was this a deliberate decision?
    - Would you do it differently at a different time / with another patient? How? Why? Can you give an example? Please elaborate.
    - What strategy have you followed?

• Which sources of information/cues that are relevant to your communication do you consult in daily practice?

- - - Feedback from colleagues? From patients? Online modules? Online databases?
    - What other information sources are you aware of?

Additional questions:

• Are there things that you are going to change in your practice based on the interview / observations today?

• What was the effect of me being present today? Did it have an affect the way you practiced? How? In which way? Please elaborate.
